# Simultaneously Recovery of Thorium and Tungsten through Hybrid Electrolysis–Nanofiltration Processes

**DOI:** 10.3390/toxics12020103

**Published:** 2024-01-26

**Authors:** Geani Teodor Man, Paul Constantin Albu, Aurelia Cristina Nechifor, Alexandra Raluca Grosu, Diana Ionela Popescu (Stegarus), Vlad-Alexandru Grosu, Virgil Emanuel Marinescu, Gheorghe Nechifor

**Affiliations:** 1 Analytical Chemistry and Environmental Engineering Department, University Politehnica of Bucharest, 011061 Bucharest, Romania; geani.man@icsi.ro (G.T.M.); aureliacristinanechifor@gmail.com (A.C.N.); andra.grosu@upb.ro (A.R.G.); 2 National Research and Development Institute for Cryogenics and Isotopic Technologies–ICSI, 240050 Râmnicu Valcea, Romania; stegarus@icsi.ro; 3 Radioisotopes and Radiation Metrology Department (DRMR), IFIN Horia Hulubei, 023465 Măgurele, Romania; paulalbu@gmail.com; 4 Department of Electronic Technology and Reliability, Faculty of Electronics, Telecommunications and Information Technology, University Politehnica of Bucharest, 061071 Bucharest, Romania; 5 Department of Physical-Chemical Tests, National Institute for Research and Development in Electrical Engineering ICPE–CA Bucharest, 030138 Bucharest, Romania; virgil.marinescu@icpe-ca.ro

**Keywords:** thorium recovery, wolfram recovery, membrane processes, membrane electrolysis, nanofiltration, thorium recycling, wolfram recycling, Pourbaix diagrams

## Abstract

The recovery and recycling of metals that generate toxic ions in the environment is of particular importance, especially when these are tungsten and, in particular, thorium. The radioactive element thorium has unexpectedly accessible domestic applications (filaments of light bulbs and electronic tubes, welding electrodes, and working alloys containing aluminum and magnesium), which lead to its appearance in electrical and electronic waste from municipal waste management platforms. The current paper proposes the simultaneous recovery of waste containing tungsten and thorium from welding electrodes. Simultaneous recovery is achieved by applying a hybrid membrane electrolysis technology coupled with nanofiltration. An electrolysis cell with sulphonated polyether–ether–ketone membranes (sPEEK) and a nanofiltration module with chitosan–polypropylene membranes (C–PHF–M) are used to carry out the hybrid process. The analysis of welding electrodes led to a composition of W (tungsten) 89.4%; Th 7.1%; O_2_ 2.5%; and Al 1.1%. Thus, the parameters of the electrolysis process were chosen according to the speciation of the three metals suggested by the superimposed Pourbaix diagrams. At a constant potential of 20.0 V and an electrolysis current of 1.0 A, the pH is varied and the possible composition of the solution in the anodic workspace is analyzed. Favorable conditions for both electrolysis and nanofiltration were obtained at pH from 6 to 9, when the soluble tungstate ion, the aluminum hydroxide, and solid thorium dioxide were formed. Through the first nanofiltration, the tungstate ion is obtained in the permeate, and thorium dioxide and aluminum hydroxide in the concentrate. By adding a pH 13 solution over the two precipitates, the aluminum is solubilized as sodium aluminate, which will be found after the second nanofiltration in the permeate, with the thorium dioxide remaining integrally (within an error of ±0.1 ppm) on the C–PHF–M membrane.

## 1. Introduction

The performances proven by membrane separation processes in various ecological and greening technologies constitute important arguments for their involvement in the recovery and recycling of various urban wastes, especially those that generate toxic products [1,2,3,4]. Thus, in order to develop a technology for the separation and valorization of some metallic elements from specific urban waste, baromembrane processes can be applied, when the elements are in a colloidal (nanometric) or suspension (micrometric) state, or processes with electric and/or concentration gradient, if the metals are found in the forms of ions [5,6,7,8].

The development of new technologies for the recovery of metals from urban waste requires solid arguments regarding the value of the separated metals or the negative impact on the environment if such metals are not separated and recycled [9,10,11]. A special case reported recently [12] shows that metals such thorium or tungsten that come from electric and electronic equipment or materials used in construction (refractory bricks and welding electrodes) can appear in urban waste [13,14]. The recovery of both metals is of technical importance because they have high practical utility [11,15] but also because they have a particularly undesirable impact on the environment, especially thorium [16].

Both thorium and tungsten are obtained from ores by established processes such as hydrometallurgical treatment [17,18,19], separation [20,21,22,23,24], and treatment of alloys [25]. Moreover, tungsten has been the focus of process engineers for its recovery from various residues through electrolytic processes and acid or basic solubilization processes [26,27,28,29,30]. On the other hand, thorium has been the focus of researchers in the field of membranes for concentration, separation or purification, especially when it comes from ores in which uranium or rare earths can also be found [1,2,3,4,5,6,7,8,9,10,11,12,13,14,15,16,17,18,19,20] (Table 1).

The interest in thorium is argued both because it is a material with huge potential for energy generation, including nuclear energy [31,32,33,34,35,36,37,38,39,40], but also because, although it is radioactive, it is the object of some domestic applications that capitalize on the resistance to high temperatures of thorium dioxide (lamps, shields, crucibles, and welding electrodes) [41] or its special refractive index (lenses, glasses, and high-resolution opto-electronic apparatus) [12]. It becomes obvious that with the use of these materials, but also because thorium has a natural abundance similar to that of lead [42], it will be found in urban industrial waste arriving in various forms on municipal integrated processing platforms [43].

This work studies the simultaneous recovery of tungsten and thorium from waste coming from the welding industry with W–Th electrodes through a hybrid technology of membrane electrolysis and nanofiltration. The membrane used in the electrolysis cell is based on sulfonated polyether–ether–ketone (sPEEK–M), and the membrane used for nanofiltration is chitosan deposited on propylene hollow fiber (C–PHF–M).

## 2. Materials and Methods

### 2.1. Reagents and Materials

All reagents and organic compounds used in the presented work were of analytical grade. Th(NO_3_)_4_·5H_2_O, KSCN, NaCl, NaOH pellets, Ca(OH)_2_, HNO_3_ (68%), H_2_SO_4_ (96%), HCl 35% suprapure, and NH_4_OH 25% (analytical grade) were purchased from Merck KGaA Darmstadt, Germany.

Aluminon, Torin, and glacial acetic acid (analytical grade, Sigma-Aldrich Chemie GmbH, Steinheim, Germany) were used in the studies.

Materials: polyether–ether–ketone (PEEK), Mw = 30,000 g/mol, ρ = 1.24 g/cm^3^ powder, and average particle size = 80 micron; chitosan, high molecular weight, Code 419419, Mw > 7000 D, from shrimp shells, ≥75% (deacetylated) (Merck KGaA, Darmstadt, Germany) (Table 2), and red band W–Th welding electrodes (ProConstruct Distribution SRL, Balotești, Romania).

The hollow fibers’ polypropylene support membranes (PHF–M) were provided by GOST Ltd., Perugia, Italy [44].

The purified water characterized by 18.2 μS/cm conductivity was obtained with an RO Millipore system (MilliQ^®^ Direct 8 RO Water Purification System, Merck, Darmstadt, Germany).

### 2.2. Procedures and Methods

#### 2.2.1. Preparation of sPEEK–M Membranes from PEEK Solution in Sulfuric Acid

**Figure 1:**
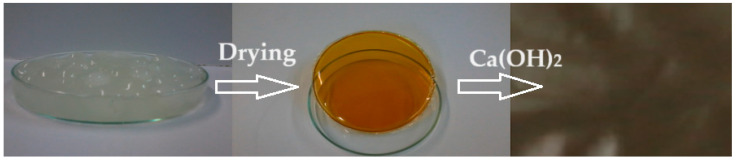
Preparation of sPEEK–M membranes from PEEK solution in sulfuric acid.

In a conical glass of 250 cm^3^, 30 mL of H_2_SO_4_ of concentration 96% is introduced, after which 2.5 g of polymer is gradually added, stirring continuously with a glass rod to avoid agglomeration of the polymer. After about 2 h of stirring, the glass is covered with a thin layer of parafilm and is kept without stirring for up to 48 h from the moment of covering the solution to favor the complete dissolution of the polymer in acid. In this time interval, sulfonation of the polymer occurs. A clear brick-colored solution with a concentration of approx. 4.5% PEEK is obtained. In order to obtain the actual support membranes, the polymer solution undergoes gelation and washing in 300 mL of water, in portions of 100 mL each, separated and applied on a spectral glass plate with the help of a chromatographic scraper knife with a slit opening between 0.5 and 0.6 mm. The pellicle is dried at 60 °C for 2 h, washed well with distilled water, and placed in a Ca(OH)_2_ bath where it is kept for 48 h, then removed and washed repeatedly in ultrapure water (Figure 1). The removal of the sulfate ion is followed by the identification reaction with a 2 mol/L barium chloride solution. The main macroscopic observations, which can be highlighted, in the case of incomplete washing are as follows [44,45]:

- Undulation of the membrane;
- The occurrence of defects (microcracks; holes);
- Local re-solubilizations (transparencies; gelations).

sPEEK–M membranes are characterized by scanning electron microscopy (SEM) and energy dispersive X-ray spectroscopy (EDAX).

#### 2.2.2. Preparation of Chitosan–Polypropylene Hollow Fiber Membranes (C–PHF–M)

**Figure 2:**
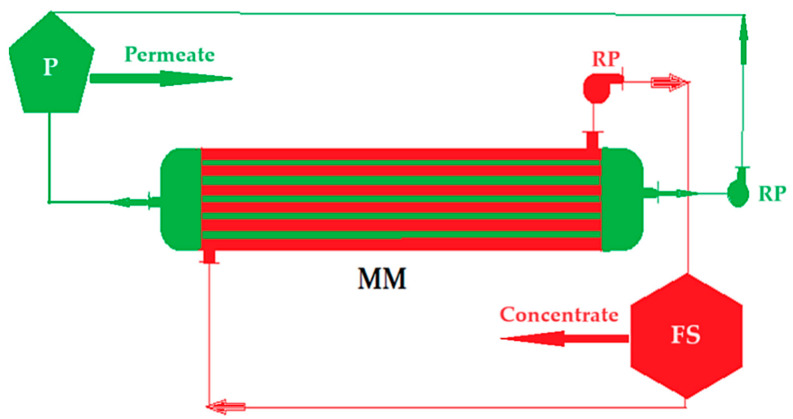
Schematic of the nanofiltration installation for the preparation of C–PHF–M membrane: MM—membrane module; P—permeate; FS—feed solution; RP—recirculation pumps.

In a round-bottomed flask, a chitosan solution is prepared under continuous magnetic stirring by dissolving 10 g of chitosan powder in 990 mL of 10% acetic acid solution. After mixing for about two hours, a yellowish-white solution is obtained, which is the feed phase of the ultrafiltration membrane module (Figure 2) equipped with a polypropylene hollow fiber membrane. With the help of a recirculation pump, a pressure of 6 bars is ensured in the space between the body of the module and the outside of the membranes in a closed circuit. The permeate, which is made up of the acetic acid solution, is collected inside the membranes. A layer of chitosan is deposited on the polypropylene hollow fiber membrane [45,46].

The C–PHF–M membranes were characterized by scanning electron microscopy (SEM) and energy dispersive X-ray spectroscopy (EDAX) and from the perspective of performance in the nanofiltration process.

#### 2.2.3. Electrolysis of Tungsten and Thorium-Based Electrodes in Acidic Aqueous Solution

**Figure 3:**
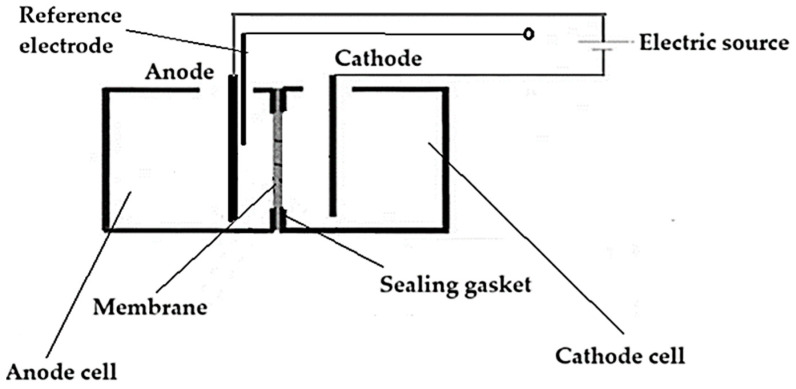
The schema of the electrolysis cell of tungsten electrodes for the recovery of thorium and tungsten.

A volume of 250 mL of each solution was introduced, in turn, into an electrolysis cell (Figure 3) of the MASTECH HY3005D-3 potentiostat equipment provided with three electrodes: tungsten welding electrode as the anode, platinum wire as the cathode, and a reference electrode. The potentiostat was operated at a potential of +20.0 V and an electrolysis current of 1.0 A. The pH in the anodic space was imposed at 0, 2, 4, 6, 8, 10, and 12, and in the cathodic space at pH 13, using hydrochloric acid and sodium hydroxide, respectively. The experimental procedure took place at room temperature. After six hours of work at the cell base, both solutions (anodic and cathodic) were collected and subjected to nanofiltration in order to recover thorium as thorium dioxide and tungsten as tungstate ion. The chloride anion concentration was followed up with a combined selective chloride electrode (HI 4107, Hanna Instruments Ltd., Leighton Buzzard, UK) and a multiparameter system (HI 5522, Hanna Instruments Ltd., Leighton Buzzard, UK).

For the recovery of thorium dioxide, both the solution from the anodic space and the one from the cathodic space are nanofiltered, both of which constitute the feed solutions (FS) of the membrane mode in the installation diagram in Figure 3. This time, the module is equipped with a chitosan–polypropylene nanofiltration membrane. A separate module is used for each of the two feeding solutions so that the components in the anodic and cathodic space can be identified.

### 2.3. Equipment

The membranes’ microscopy studies, SEM and HFSEM, were performed on a Hitachi S4500 system (Hitachi High-Technologies Europe GmbH, Krefeld, Germany) [46].

The UV-Vis analyses of the solutions were performed on a Spectrometer CamSpec M550 (Spectronic CamSpec Ltd., Leeds, UK) [47].

The UV-Vis studies on the nanoparticle samples’ composition were performed on dual-beam UV equipment—Varian Cary 50 (Agilent Technologies Inc., Santa Clara, CA, USA) at a resolution of 1 nm, spectral bandwidth of 1.5 nm, and a 300 nm/s scan rate. The UV-Vis spectra of the samples were recorded for a wavelength from 200 to 800 nm, at room temperature, using 10 mm quartz cells [48].

MASTECH HY3005D-3 (San Jose, CA, USA) is a working power supply which has an electrical output, continuously adjustable, between 0–30 V DC and 0–5 A.

The validation of the electrochemical processes was followed up with a PARSTAT 2273 Potentiostat (Princeton Applied Research, AMETEK Inc., Oak Ridge, TN, USA). A glass cell with three electrodes set up was used [47,48].

The pH of the medium was followed up with a combined selective electrode (HI 4107, Hanna Instruments Ltd., Leighton Buzzard, UK) and a multiparameter system (HI5522, Hanna Instruments Ltd., Leighton Buzzard, UK) [49].

To assess and validate the content in metal ions, the atomic absorption spectrometer AAnalyst 400 AA Spectrometer (Perkin Elmer Inc., Waltham, MA, USA) with WinLab32–AA software version 6.0 or above (Perkin Elmer Inc., Waltham, MA, USA), with a single-element hollow cathode lamp, was used [50].

In the case of the present work, for the validation of the SEM and EDAX analyses, the tungsten electrode samples subjected to the analysis were visualized with the help of the FESEM–FIB workstation (scanning electron microscope with field emission electron and focused beam of ions) model Auriga (Carl Zeiss SMT, Oberkochen, Germany) by means of the secondary electron/ion detector (SESI) in the sample chamber for the topography/morphology of the surface of the analyzed samples [51].

The verification of the chemical composition was carried out with the help of the EDS probe (energy dispersive spectrum for the characteristic X-ray) produced by Oxford Instruments, UK—energy dispersive spectrometer model X–MaxN with the Aztec acquisition and processing software integrated on the FESEM–FIB Auriga working station [52].

Secondary electron (SE) topography images were acquired and analyzed from the sample chamber with the Everhart Thornley SESI secondary electron detector with the Faraday cup (SESI)/in-column annular SE in-lens detector, at a voltage acceleration of 5 kV for sample visualization and 10 kV for EDS spectroscopy, for the following types of analyses carried out by the energy dispersive probe on the surface of the analyzed sample:

- Semi-quantitative spot analyses located at certain intervals in the same micro-area for the distribution of elements from the point of view of composition on the surface of the material as well as the variational verification of the composition of the investigated micro-area with the points from which the respective spectra were acquired.
- Elemental mapping analyses, i.e., obtaining spectral images—where the distribution of the elements on the surface swept by the electron beam is superimposed and possible compositional differences are highlighted—increasing the area of the present element or the appearance/disappearance of an analyzed and identified element.
- The extractive spectrophotometric analysis of thorium was performed on the CamSpec M550 spectrophotometer and validated on the Varian Cary 50 using the established methods and techniques [53,54,55].

## 3. Results and Discussion

The present work is part of the trend of removal, recovery, recycling, and valorization of metals and metal ions with a severe impact on the environment through various techniques that include membrane [56].

The approach to the valorization of thorium and tungsten from welding electrodes is an example of the use of hybrid processes using membranes.

### 3.1. Characterization of the sPEEK–M Membrane

**Figure 4:**
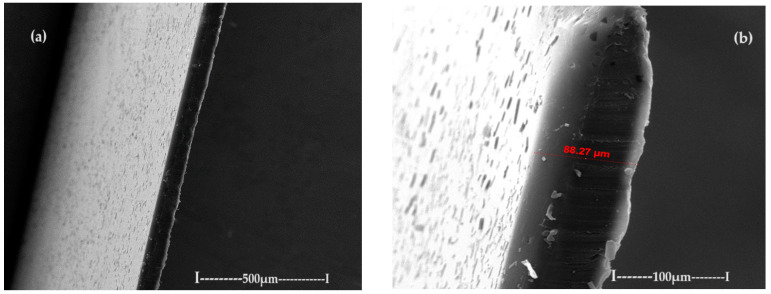
The images obtained by scanning electron microscopy (SEM) for (**a**) the section of the membrane after keeping it in the calcium hydroxide bath; and (**b**) the detail of the section.

**Figure 5:**
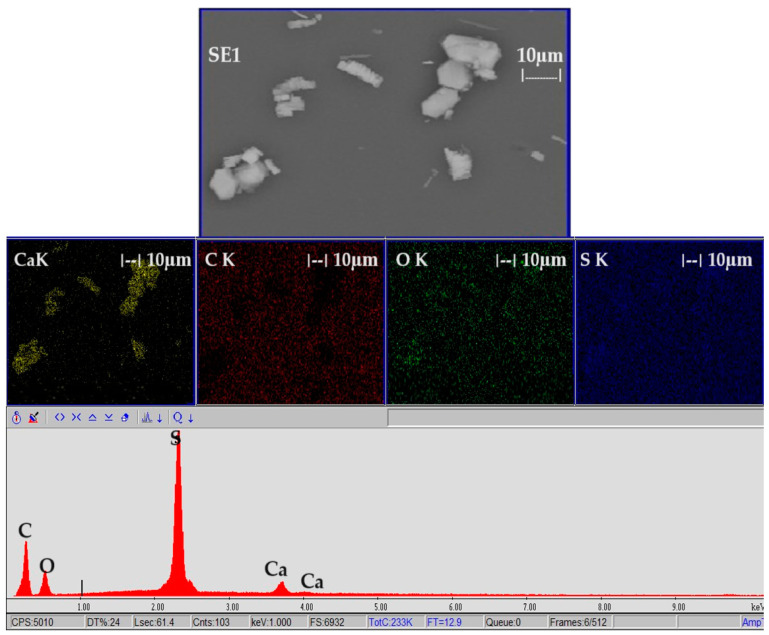
Energy dispersive X-ray spectroscopy (EDAX) of the membrane after keeping it in the calcium hydroxide bath.

**Figure 6:**
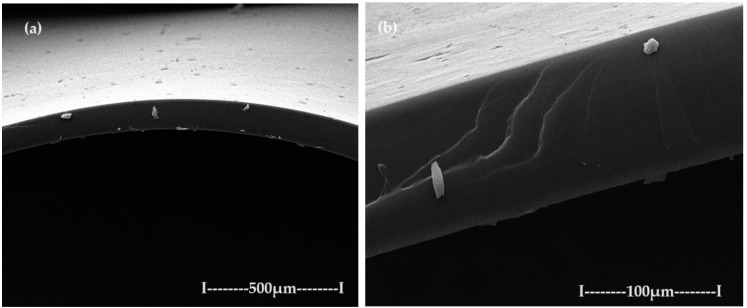
The image obtained by scanning electron microscopy (SEM) for (**a**) the section of the washed membrane; and (**b**) the detail of the section.

**Figure 7:**
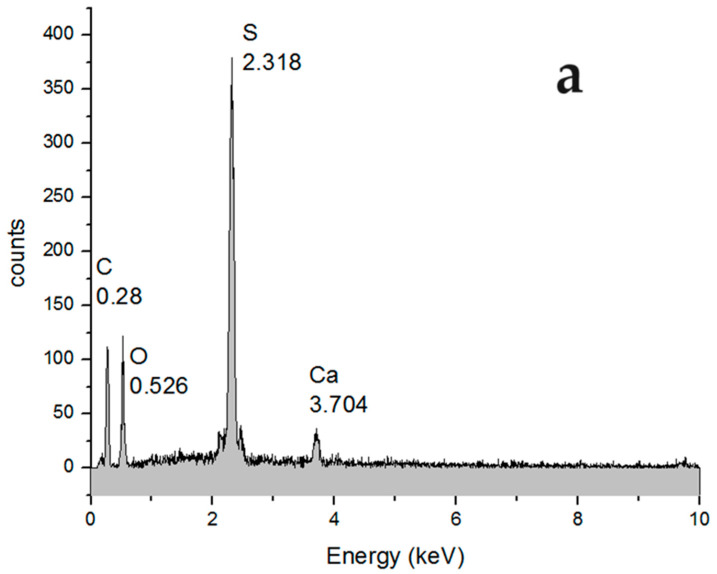

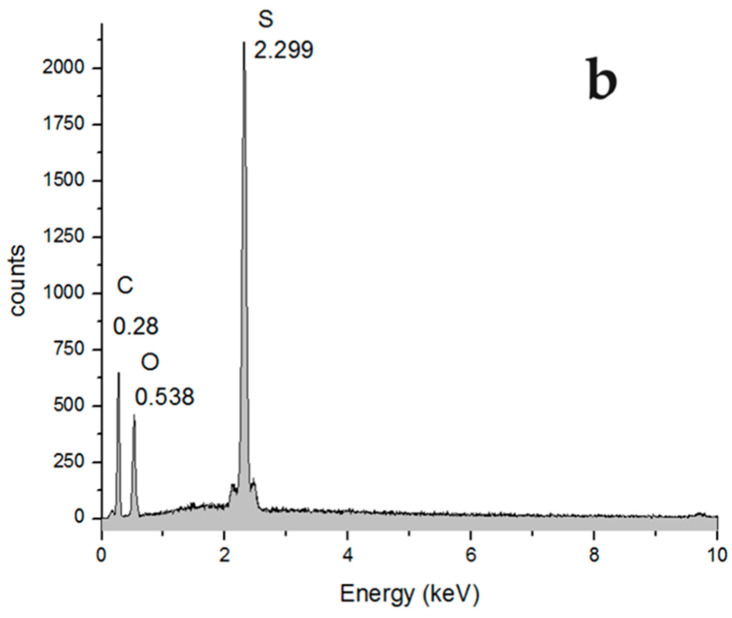
Comparative energy dispersive X-ray spectroscopy (EDAX): (**a**) membrane before washing; and (**b**) membrane after keeping it in ultrapure water.

Membranes of sulfonated polyether–ether–ketone (sPEEK) [44,45] or sPEEK polymer composites [46] are used in ultrafiltration, nanofiltration, or reverse osmosis processes [44]. Three samples of 1 cm^2^ each from the membrane obtained after fixing with calcium hydroxide and after being kept in ultrapure water are introduced into a Dewar vessel with liquid nitrogen and fractured. After coating with a superficial layer (50 nm) of gold, the membrane obtained from the fixing bath with calcium hydroxide (Figure 4 and Figure 5) and the one obtained after washing and maintenance in pure water (Figure 6 and Figure 7) are SEM and EDAX characterized.

### 3.2. Characterization of C–PHF–M Membrane

**Figure 8:**
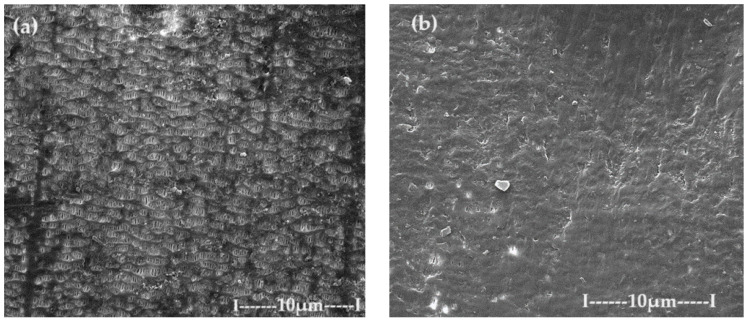
The image obtained by scanning electron microscopy (SEM) for (**a**) the polypropylene hollow fiber membrane (PHF–M) surface; (**b**) chitosan–polypropylene hollow fiber membrane (C–PHF–M).

**Figure 9:**
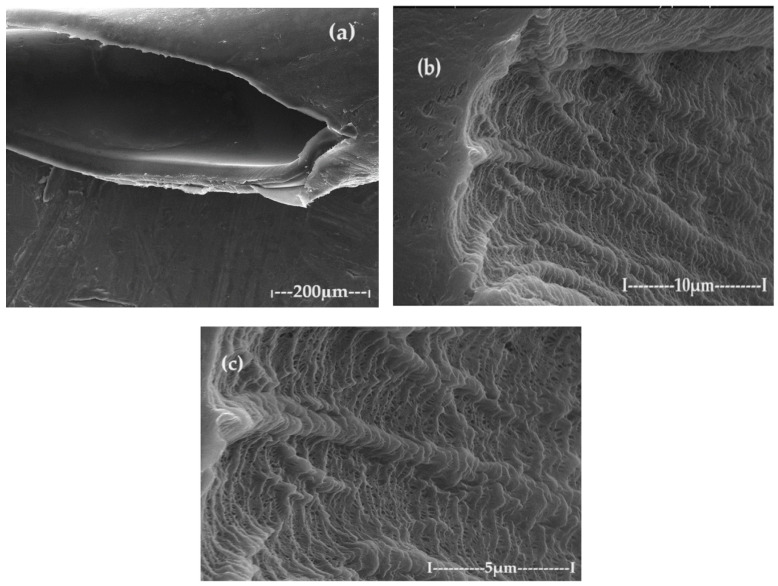
The image obtained by scanning electron microscopy (SEM) of the chitosan–polypropylene hollow fiber membrane (C–PHF–M) section: (**a**) general section; (**b**) detail of the section highlighting the active layer; and (**c**) detail of the polypropylene hollow fiber membrane (PHF–M) substrate.

The membranes based on chitosan have been frequently used to carry out membrane processes for the separation of ions and molecules [57,58,59,60], but in this work, chitosan membranes are obtained on a polypropylene hollow fiber (C–PHF–M) support. Practically, the polypropylene hollow fiber (PHF) support, which has previously presented ultrafiltration performances [44,45,46], is transformed in nanofiltration composite membranes (C–PHF–M) by ultrafiltration of a chitosan solution to obtain in situ a selective layer of chitosan. Samples of 3 cm of the polypropylene hollow fiber membrane and the chitosan–polypropylene hollow fiber membrane were fractured in liquid nitrogen and covered with a superficial layer (50 nm) of gold and characterized by scanning electron microscopy (SEM) (Figure 8 and Figure 9).

The support membrane (Figure 8a) with pores of 0.002–0.2 µm specific for micro and ultrafiltration is transformed after the coating with chitosan (Figure 8b) into the composite membrane (C–PHF–M) for nanofiltration with pores smaller than 0.002 µm.

### 3.3. Electrolysis of Tungsten Electrodes for the Recovery of Thorium and Tungsten

The raw material required for the hybrid thorium recovery process is the remains and ends of the welding electrodes at high temperatures. These materials can be selectively collected on construction sites or industrial production halls and undergo membrane electrolysis followed by nanofiltration, when thorium is recovered as thorium dioxide and tungsten as a tungstate solution.

#### 3.3.1. SEM and EDAX Analysis of Welding Electrodes

**Figure 10:**
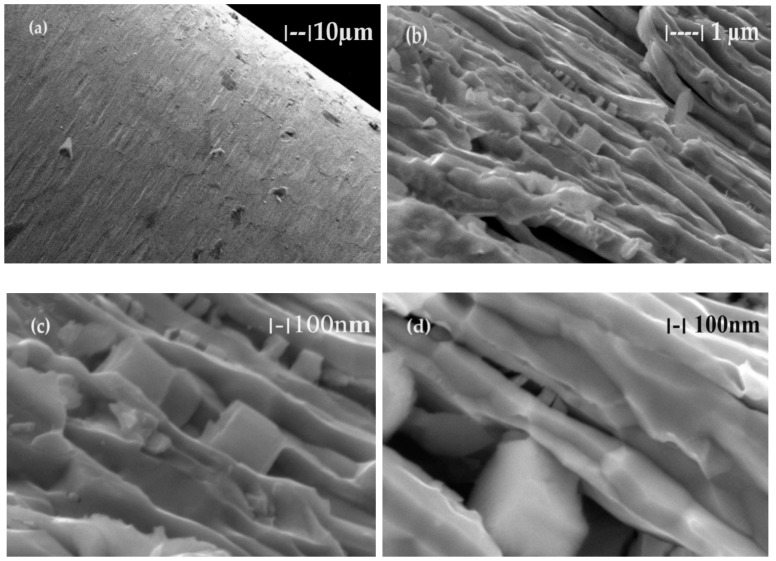
Images obtained by scanning electron microscopy (SEM) for (**a**) the electrode surface; (**b**) the section of the electrode 20,000×; (**c**) the section of the electrode 50,000×; and (**d**) the detail of the electrode section 50,000×.

**Figure 11:**
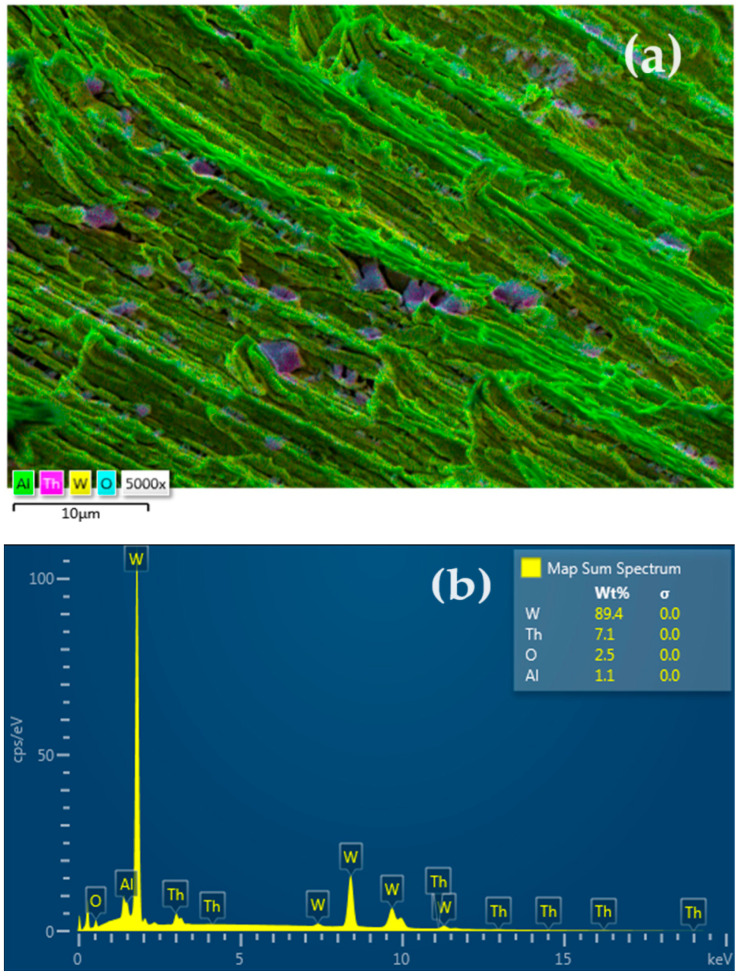

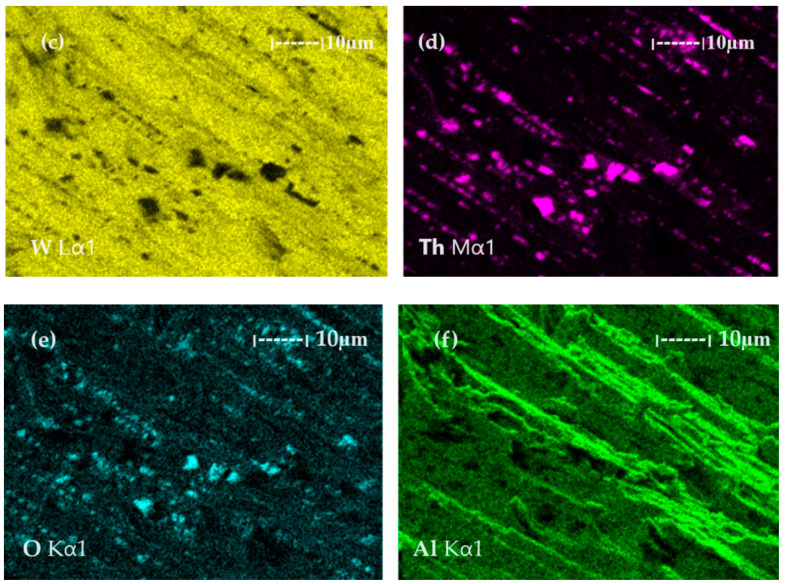
The elemental distribution obtained by energy dispersive X-ray spectroscopy (EDAX) for the fracture of the welding electrode: (**a**) general distribution of the elements in colors; (**b**) quantitative elemental composition; (**c**) tungsten distribution; (**d**) thorium distribution; (**e**) oxygen distribution; and (**f**) aluminum distribution.

The SEM and EDAX characterization of the raw material, the welding electrodes, used in this work highlighted the morphology but also the surprising composition (Figure 10 and Figure 11). Macroscopically, the welding electrode has a relatively smooth surface (Figure 10a), but with high probability, and tungsten filaments appear in the section (Figure 10b), joined by thorium dioxide cubes (Figure 10c,d).

In order to confirm the assessments made by analyzing the electron microscopy, energy dispersive X-ray spectroscopy (EDAX) analysis was performed in the electrode fracture (Figure 11). Surprisingly (Figure 11a,b), apart from tungsten, thorium and oxygen appear in the fracture, as well as a measurable amount of aluminum.

The elemental distribution (Figure 11c,d,f) confirms the assumption that tungsten forms filaments and plates that are joined with nanometric cubes of thorium dioxide. Aluminum, which is distributed over the entire fracture surface of the electrode (Figure 11f), mainly covers the tungsten filaments and plates, suggesting that it is an element with a binding role. The quantitative composition (Figure 11b) is W 89.4%; thorium 7.1%; oxygen 2.5%; and aluminum 1.0%.

#### 3.3.2. Membrane Electrolysis of Welding Electrodes

**Figure 12:**
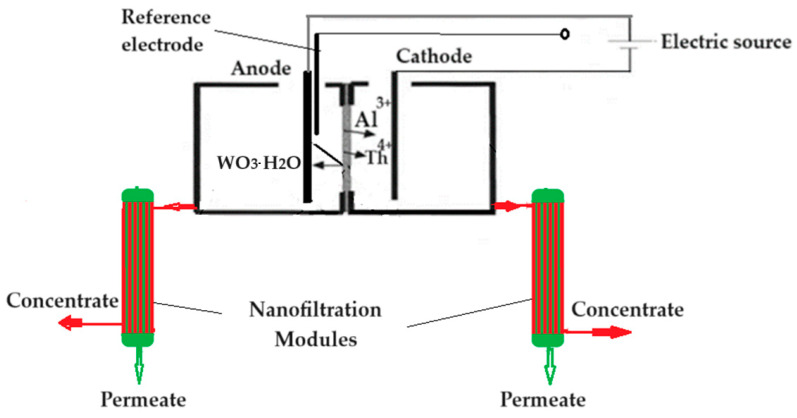
Scheme of the hybrid installation for membrane electrolysis and nanofiltration of tungsten electrodes.

In order to avoid the loss of thorium in the form of thorium dioxide of nanometric dimensions from the membrane electrolysis process, this process was coupled with the nanofiltration of both the solution from the cathodic space and the solution in the anodic space (Figure 12).

The probable reactions in the anodic space (1–4) and cathodic space (5,6) are as follow:

Anode

W + 12H_2_O ⇌ WO_4_^2−^ + 8H_3_O^+^ + 6e^−^ (1)

Th + 6H_2_O ⇌ ThO_2_ + 4H_3_O^+^ + 4e^−^ (2)

Al ⇌ Al^3+^ + 3e^−^ (3)

3H_2_O ⇌ 2H_3_O^+^ + 2e^−^ + 1/2 O_2_ (4)

Cathode

2H_2_O + 2e^−^ ⇌ H_2_ + 2HO^−^ (5)

2H_3_O^+^ + 2e^−^ ⇌ H_2_ + 2H_2_O (6)

**Figure 13:**
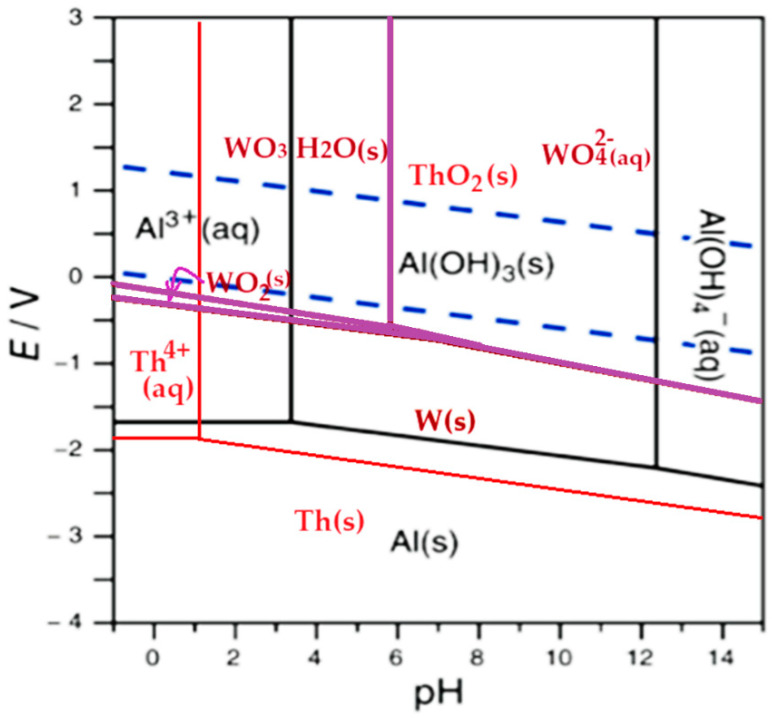
Superimposed Pourbaix diagrams of tungsten (purple), thorium (red), and aluminum (black), with the blue dotted lines delimiting the stability domain of water.

However, the choice of the potential of the working electrode (anode) as well as the pH in the anodic space largely determine the speciation of each component element of the welding electrodes considered in this study: tungsten, thorium, and aluminum (Table 3 and Figure 13). Thus, by superimposing the Pourbaix diagrams [61,62,63] of the three elements (Figure 13), it is possible to specify, at constant potential, the chemical species for each pH in the anodic space (Table 3).

At the potential of 20.0 V and pH = 0 in the anodic space, and pH = 13 in the cathodic space, following electrolysis, tungsten will be found in the form of tungstic acid WO_3_·H_2_O(s), thorium as Th^4+^(aq), and aluminum as Al^3+^(aq) (Figure 13). The two cations, Th^4+^ and Al^3+^, will migrate to the cathode, and the tungstic acid will remain in the anodic space. Tungsten in the anodic space will be recovered by nanofiltration through the module attached to this space. Thorium will be immobilized as a solid in the cathodic space in the form of thorium dioxide (ThO_2_), and the aluminum ion will transform into sodium aluminate (AlO_2_^−^), a soluble. Through nanofiltration [64,65,66] of the solution from the cathodic space through the module attached to this space, thorium dioxide will be retained on the membrane (concentrate) and aluminate ion as the permeate.

At the same potential value of the anodic space, but at pH between 1 and 3.5, the chemical species of the three elements will be tungsten as WO_3_ H_2_O(s), thorium as ThO_2_(s), and aluminum as Al_3_^+^(aq). In this case, the solution in the anodic space is nanofiltered after treatment with ammonia solution, and ammonium tungstate will pass into the permeate and thorium dioxide will remain on the membrane in the concentrate. The solution in the cathodic space will contain sodium aluminate, which, being in solution, does not require nanofiltration. However, for the safety of the hybrid process, the solution from the cathodic space also undergoes nanofiltration, from which we will find sodium aluminate in the permeate and any other solid impurity will be retained on the membrane.

If the anode has a potential of 20.0 V and the pH in the anodic space stays between 3.5 and 6, the chemical species obtained for the three elements are tungsten as WO_3_·H_2_O(s), thorium as ThO_2_(s), and aluminum as Al(OH)_3_ (s). In this case, the solutions from the anodic and cathodic spaces obtained after electrolysis are mixed together and subjected to nanofiltration, and thorium dioxide is retained in the concentrate and the tungstate and aluminate ions are found in the permeate. To separate the two anions, the pH is adjusted to 5 when the aluminum hydroxide precipitates, which is retained by nanofiltration in the second module of the installation.

If the anode has a potential of 20.0 V, and the pH in the anodic space is kept between 6 and 12, then the chemical species obtained for the three elements are tungsten as WO_4_^2−^(aq), thorium as ThO_2_(s), and aluminum as Al(OH)_3_(s).

In this case, the nanofiltration of the anodic space takes place in two stages:

- The first stage is at the pH (6–12) in this space when a permeate, containing the soluble tungstate ion, and a concentrate, containing thorium dioxide and aluminum hydroxide, are obtained;
- The second stage is when the concentrate is mixed with the pH 13 solution from the cathodic space, the aluminum is solubilized as aluminate, passing into the permeate, and thorium dioxide remains in the concentrate.

If the anode has a potential of 20.0 V, and the pH in the anodic space is above 12, then the chemical species obtained for the three elements are tungsten as WO_4_^2−^(aq), thorium as ThO_2_(s), and aluminum as AlO_2_^−^(aq).

In this case, the nanofiltration of the anodic space takes place in two stages:

- The first stage is at the pH in this space (above 12) when a permeate containing soluble tungstate and aluminate ions is obtained, as well as a concentrate containing thorium dioxide and aluminum hydroxide;
- The second stage is when the permeate is brought to a pH between 6 and 9, and the aluminum precipitates as hydroxide and is separated from the tungstate ion by nanofiltration in the second module.

Tungsten recovery after electrolysis means nanofiltration of the solution in order to purify it, because it is obtained as tungstate either by neutralizing tungstic acid or directly, at a pH higher than 6.

The recovery of thorium requires additional attention, firstly because thorium is a radioactive element, and secondly because being obtained as a thorium dioxide dispersion, it will have to be nanofiltered under optimal conditions so as not to be lost in the permeate.

**Figure 14:**
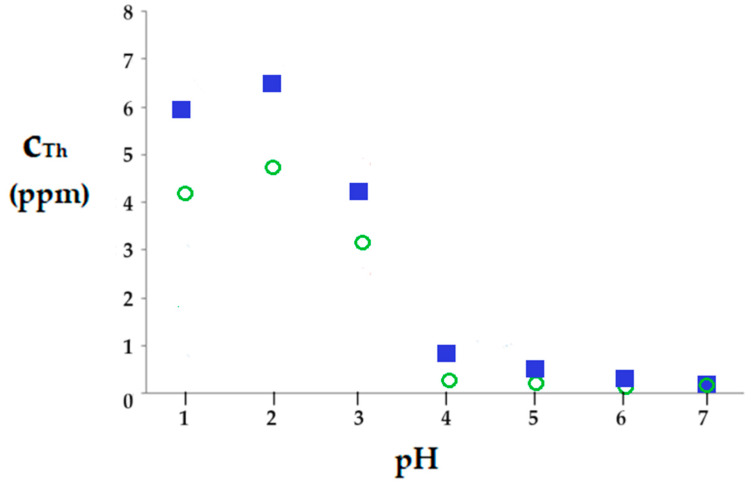
Variation in thorium concentration in the permeate during nanofiltration of thorium dioxide nanodispersion, depending on pH: (green) thorium dioxide obtained from the electrolysis process of the welding electrode; and (blue) thorium dioxide obtained by precipitation from the solution.

The results obtained during the nanofiltration of nanodispersions containing thorium dioxide, depending on the pH and at an 8-bar working pressure, show flows between 8.5 and 14 L/m^2^·h and a removal of thorium reaching below 1 ppm in the permeate, at pH higher than 4, with a determination error of ±0.1 ppm (Figure 14). This concentration of thorium is below the natural background in various locations in the world, especially those where rare earths are also found [67,68,69,70,71,72].

The higher concentration of thorium in the permeate at pH below 3 is due to the existence of thorium dioxide in dispersion and as Th^4+^ ion, which is partially retained by the C–PHF–M membrane, which is in cationic form (^+^H_3_N–R). At pH above 3, the concentration of thorium in the permeate drops sharply to 0.1 ppm, which shows that thorium is found almost entirely as thorium dioxide ThO_2_, and any thorium ions are retained by the membrane in cationic form.

What is interesting is the evolution of the permeate concentration during nanofiltration depending on the morphological nature of thorium dioxide (Figure 13). Thus, over the entire pH range, the concentration of thorium in the permeate for thorium dioxide obtained from electrolysis (Figure 14—green circles) is lower than that of the same element precipitated from the Th(NO_3_)_4_ solution (Figure 14—blue squares).

**Figure 15:**
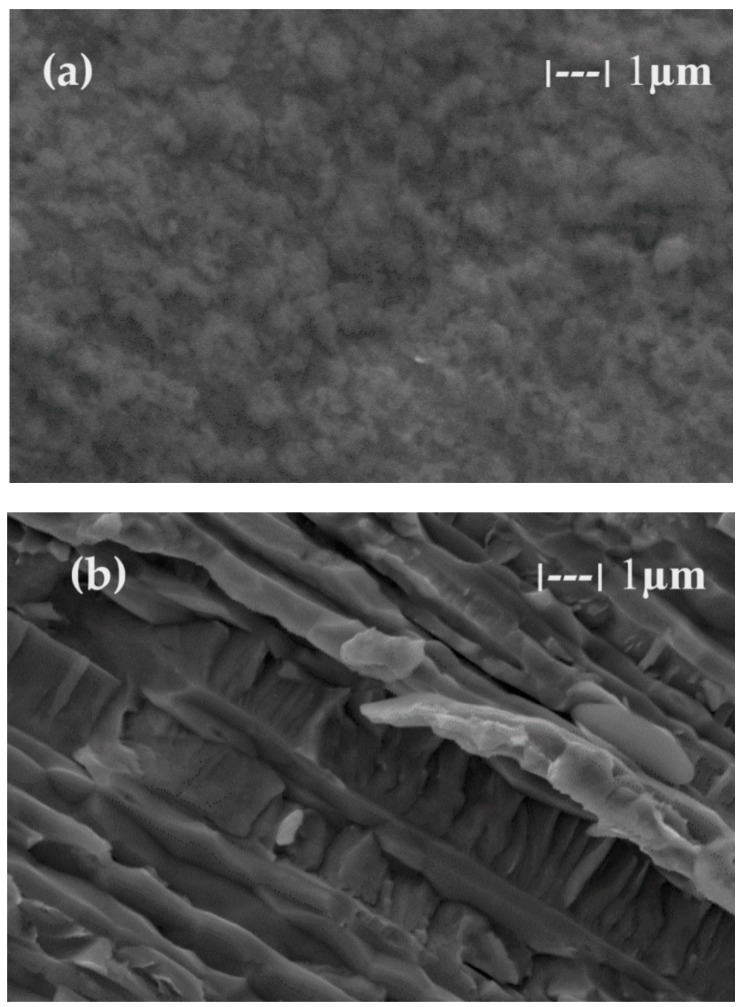
The morphological aspect highlighted through scanning electron microscopy (SEM) for thorium dioxide obtained by (**a**) precipitation of Th(NO_3_)_4_; (**b**) electrolysis of the welding electrode.

There can be two reasons for this behavior:

- The existence of thorium in ionic form and as thorium dioxide of different morphology (Figure 15);
- The ionic charge of the nanofiltration membrane.

The first argument would probably have a greater influence on the thorium concentration in the permeate. Thus, the thorium dioxide obtained by the precipitation of Th(NO_3_)_4_ in amorphous form (Figure 15a) is retained to a lesser extent on the membrane than the crystalline form of thorium dioxide released from the interstices of the tungsten wires (plates) through electrolysis of the welding electrode (Figure 10c,d and Figure 15b).

According to the results presented in Figure 14 and using Formula (7) for the recovery rate (RR) of thorium, it falls between the values of 97.71% and 99.96%.

(7) $$RR\left(\%\right)=\frac{\left(c_{0}-c_{f}\right)}{c_{0}}\cdot 100$$

where *c_f_* is the final concentration of the solute (considered chemical species, Th) and *c*_0_ is the initial concentration of solute (considered chemical species, Th).

**Figure 16:**
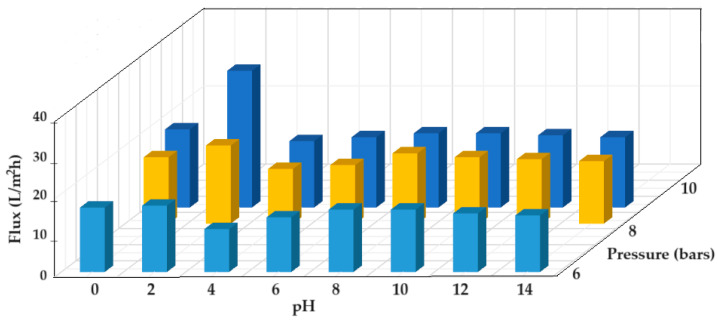
Variation in the nanofiltration flow of nanodispersions containing thorium dioxide, depending on pH and the working pressure.

The permeate flow performances of nanofiltration of thorium dioxide nanodispersions from the complex system obtained by electrolysis, depending on pH and working pressure, are presented in Figure 16.

Over the entire pH range (Figure 16), the fluxes increase with increasing operating pressure from 6 to 10 bar. Each time, from pH 0 to 2, the values of the permeate flows increase up to about 30 L/m^2^h, which is obtained at 10 bars. Starting at pH 4, the flow values increase slightly until pH 10, after which there is a slight decrease.

The recorded flux results are consistent with the speciation of thorium and of the C–PHF–M membrane. At low pH, below 2, thorium is found as ion, and after pH 2, thorium dioxide is formed. It is likely that the size of thorium dioxide nanoparticles increases with the pH, which improves filterability. However, after pH 8, the viscosity of the alkaline solution causes a slight decrease in the permeate flow.

From the point of view of the nanofiltration results, the pH in the electrolysis installation can be up to 2 when we have thorium ions and tungstic acid in the solution, or between 8 and 10 when we have thorium dioxide and tungstate ions in the solution.

The presence of aluminum in the system, even in traces, could impose electrolysis with the anodic space at strongly alkaline pH when aluminum is found as aluminate, tungsten as tungstate, both in solution, and thorium as an insoluble precipitated form of thorium dioxide. In the latter case, the separation of the anodic and cathodic spaces through the sPEEK membrane becomes unnecessary, the recovery of thorium dioxide being achieved through the first nanofiltration process. The separation of aluminum is achieved through the second nanofiltration process, after adjusting the pH to between 5 and 9, when the aluminum is retained on the membrane in the form of insoluble aluminum hydroxide.

## 4. Conclusions

The recovery and recycling of metals that generate toxic ions in the environment is of particular importance, especially when these are tungsten, aluminum and, above all, thorium.

This paper presents the separation and recovery of tungsten, thorium, and aluminum from welding electrodes at high temperature in a hybrid installation of membrane electrolysis and nanofiltration. The membranes used are made from sPEEK for the separation of anodic and cathodic spaces in the electrolysis cell, and from chitosan deposited on a polypropylene hollow fiber membrane (C–PHF–M) in the nanofiltration module.

Through electrolysis at 20.0 V and variable pH, the speciation of the three studied elements could be separated. Favorable conditions for both electrolysis and nanofiltration were obtained at pH from 6 to 9, when the soluble tungstate ion, solid aluminum hydroxide, and solid thorium dioxide were formed. Through the first nanofiltration process, the tungstate ion was obtained in the permeate, and thorium dioxide and aluminum hydroxide in the concentrate. By adding a pH 13 solution over the two precipitates, aluminum was solubilized as soluble sodium aluminate, which, after the second nanofiltration process, was found in the permeate, the thorium dioxide remaining integrated (within an error of ±0.1 ppm) in the C–PHF–M membrane.

Variants of the operation procedure of the hybrid electrolysis and nanofiltration installation were suggested by superimposing the Pourbaix diagrams of tungsten, thorium, and aluminum.

The increase in the working potential above the value of 20.0 V causes only an increase in molecular oxygen in the anodic space as a chemical speciation modification.

## Figures and Tables

**Table 1:** The main techniques and methods of obtaining tungsten and thorium and their applications.

Element	Techniques, Methods, or Applications	Refs.
Tungsten	Utilization of tungsten residue	[1]
	Tungsten resources and potential extraction	[3]
	Recovery of W(VI) from wolframite ore using new synthetic Schiff-based derivative	[17]
	Extraction of sodium tungstate from tungsten ore	[18]
	Progress in sustainable recycling and circular economy of tungsten carbide	[19]
	Tungsten resources and potential extraction from mine waste	[20]
Thorium	Recovery and transport of thorium (IV) through polymer inclusion membrane	[9]
	Process for the separation of U(VI), Th(IV) from rare earth elements by using ionic liquid Cyphos IL 104.	[11]
	Thorium removal, recovery, and recycling	[12]
	Polymeric materials for rare earth element recovery	[13]
	Highly efficient adsorbent to remove thorium ions	[15]
	Impacts of uranium- and thorium-based fuel cycles with different recycle options	[16]

**Table 2:** The characteristics of the used polymer compounds.

Organic Compounds	Name and Symbol	Molar Mass (g/mol)	Solubility (g/L)	pKa
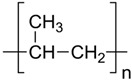	Polypropylene (PP)	–	–	7.0
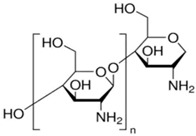	Chitosan (Chi)	High molecular weight (up to 7000 D)	Soluble in acid media (0.5 M HCl: 50 mg/mL)	6.2 to 7.0
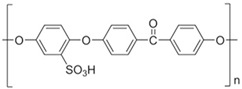	Sulfonated polyether–ether–ketone (sPEEK)	35,000	Organic polar solvents	2.0 to 2.2

**Table 3:** The speciation of tungsten, thorium, and aluminum elements depending on the potential and the variable pH in the anodic space, and pH 13 in the cathodic space.

Metallic Element or Membrane Reactive Group	Metal Element Speciation at Variable pH in the Anodic Space, at pH 13 in the Cathodic Space and 20.0 V Anodic Potential				
	0–1	1–3	3–6	6–12	>12
Wolfram	WO_3_·H_2_O(s)	WO_3_·H_2_O(s)	WO_3_·H_2_O(s)	WO_4_^2−^(aq)	WO_4_^2−^(aq)
Thorium	Th^4+^(aq)	ThO_2_(s)	ThO_2_(s)	ThO_2_(s)	ThO_2_(s)
Aluminum	Al^3+^(aq)	Al^3+^(aq)	Al(OH)_3_(s)	Al(OH)_3_(s)	AlO_2_^−^(aq)
sPEEK–M *	HSO_3_–Ar	HSO_3_–Ar	^−^SO_3_–Ar	^−^SO_3_–Ar	^−^SO_3_–Ar
C–PHF–M **	^+^H_3_N–R	^+^H_3_N–R	^+^H_3_N–R	H_2_N–R	H_2_N–R

* Ar—aromatic group; ** R—alkyl group.

## Data Availability

Data are contained within the article.
